# Rubisco and its regulation—major advances to improve carbon assimilation and productivity

**DOI:** 10.1093/jxb/erac475

**Published:** 2023-01-10

**Authors:** Elizabete Carmo-Silva, Robert E Sharwood

**Affiliations:** Lancaster Environment Centre, Lancaster University, UK; Hawkesbury Institute for the Environment, Western Sydney University, Richmond NSW, 2753, Australia

**Keywords:** Carboxylation, chloroplast, crop improvement, dynamic environments, metabolic regulation, photosynthesis, protein biochemistry, Rubisco, Rubisco activase

**Fig. 1.**
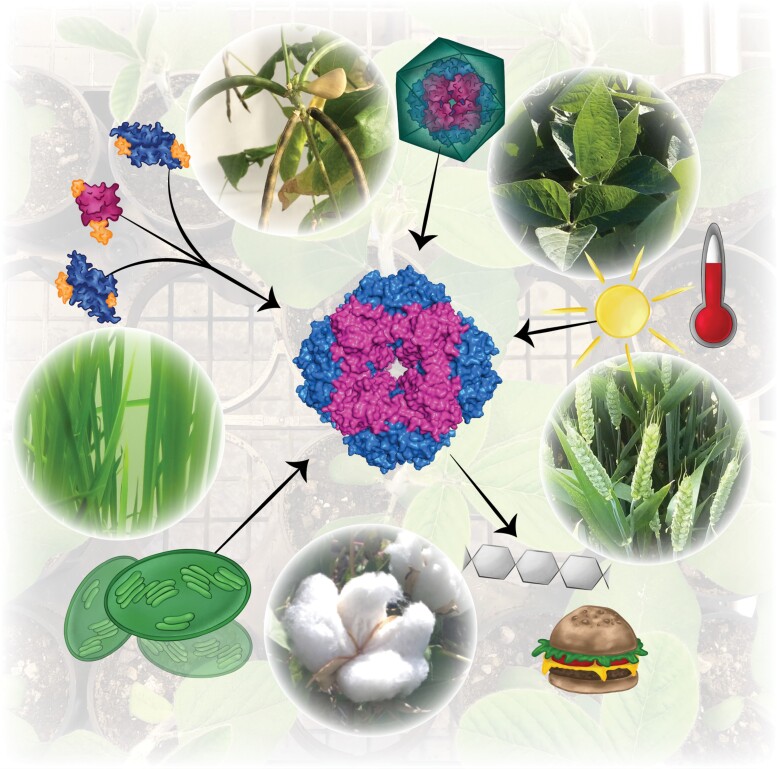
Rubisco plays a central role in biology. The carboxylation of RuBP by Rubisco initiates the Calvin-Benson-Bassham cycle and leads to the production of carbohydrates that are used for plant growth and to produce food, fuel, fiber and fodder. Its assembly is coordinated by a number of auxiliary proteins and its activity is regulated by interaction with multiple chloroplast components in response to environmental stimuli. Not only does Rubisco generate food products indirectly, but recent developments have also unlocked the potential to use Rubisco itself as a protein source for food. The Rubisco drawing is a visualisation based on protein data bank coordinates 8RUC for spinach Rubisco. Image by Daria Chrobok.

**Significant advances in Rubisco research over the past decade have highlighted the intricate nature of the CO**_**2**_ **-fixing enzyme and the complexity of environmental and cellular factors that affect its activity in photosynthetic organisms. This special issue offers comprehensive coverage of all things Rubisco (** **Fig. 1** **), from functional diversity to folding and assembly, *in vivo* regulation of its activity, including the role of its molecular partner, Rubisco activase (Rca), as well as sugar phosphate derivatives that inhibit activity.**

Since the discovery of the metabolic components of the C_3_ Calvin–Benson–Bassham Cycle (CBBC), research efforts have built the understanding of the importance of the reduction of carbon to produce carbohydrates. Ultimately, these carbohydrates are crucial for the maintenance of plant growth and yield. One of the key enzymes of the CBBC is Rubisco (ribulose-1,5-bisphosphate carboxylase), which catalyses the fixation of atmospheric carbon from CO_2_ to the sugar phosphate, RuBP (ribulose-1,5-bisphosphate). This reaction is complex and is comprised of five partial reactions to produce two molecules of 3-phosphoglycerate (Sharkey, 2023). These are then cycled through the CBBC to produce triose phosphate, the building blocks of carbohydrate, or used to regenerate RuBP to sustain carbon assimilation.

Ellis (1979) proposed Rubisco as the most abundant protein on earth, at a time when the role of chaperonin in folding Rubisco was barely understood. Rubisco has since attracted significant research efforts in understanding catalytic diversity and opportunities for improving catalysis, such as by exploring diversity in Rubiscos from a variety of photosynthetic organisms (Oh *et al*., 2023). Although the large subunit of Rubisco, which harbours the catalytic site, has long been thought to determine its catalytic properties, the important role played by the small subunit is now also recognized (Mao *et al*., 2023). The potential for small subunits to impact activity is further highlighted by work showing temperature-induced changes in small subunit expression that influence Rubisco catalysis (Cavanagh *et al*., 2023). In recent times, substantial discoveries have revealed the folding pathway for Rubisco within higher plant chloroplasts. We now know that this requires chaperonin 60/20/10 complexes and specific auxiliary factors such as Raf1, Raf2, BSDII, and RbcX (Bracher *et al*., 2017). The transplantation of this pathway into *Escherichia coli* enabled higher plant Rubisco assembly (Aigner *et al*., 2017), which will provide opportunities for directed evolution and subunit replacement to improve catalysis (Buck *et al*., 2023).

The catalysis of Rubisco is further complicated due to the requirement for non-substrate CO_2_ binding of a catalytic site lysine at position 201 (Badger and Sharwood, 2023). This forms a carbamate which is stabilized by binding of Mg^2+^, and the catalytic site is then primed for catalysis. Carbamylation of the catalytic site is crucial within the reaction mechanism for the abstraction of the proton from RuBP to form the enediol transition state intermediate. When the light reaching the leaves at the top of crop canopies decreases, carbamylation of Rubisco declines within a few minutes, reducing carbon assimilation (Taylor *et al*., 2022). During longer periods of exposure to low light or in darkness, the leaves of most species accumulate 2-carboxy-d-arabinitol 1-phosphate (CA1P), which binds tightly to carbamylated catalytic sites and inhibits Rubisco activity (Orr *et al*., 2023).

Removal of sugar phosphate derivatives such as CA1P from inhibited catalytic sites requires the action of the AAA+ protein Rca, discovered by Salvucci *et al*. (1985), which modulates activation and activity of Rubisco. Remarkably, Rca is thermolabile and, at elevated temperatures, Rubisco deactivates. However, Rca isoforms present within a single species as well as in species adapted to diverse environments have been shown to be active at warmer temperatures (Qu *et al*., 2023). As suggested, a combination of Rca and Rubisco optimizations is likely to be required to adapt crops and sustain food production in future warmer climates.

The conundrum of Rubisco research was the finding that O_2_ could also be fixed to the substrate RuBP to produce one molecule each of 2-phosphoglycolate and 3-phosphoglycerate (Sharkey, 2023). Oxygenation is an Achilles’ heel of Rubisco due to the high atmospheric concentrations of O_2_, and the photorespiratory pathway it initiates is heavily regulated in response to dynamic environmental conditions (Fu and Walker, 2023). During the history of Earth, periods of decline in atmospheric CO_2_ have led to the development of carbon-concentrating mechanisms. These vary in biochemical and anatomical features, but all ultimately enable suppression of photorespiration by increasing the CO_2_ concentration around Rubisco. This CO_2_-rich environment enabled the evolution of faster Rubiscos in C_4_ plants, algae, and cyanobacteria (Sharwood, 2017). Rubisco condensation within pyrenoids and carboxysomes by liquid–liquid phase separation has been a major step forward in understanding Rubisco aggregation, mediated by disordered linkers such as EPYC1 in *Chlamydomonas* (Ang *et al*., 2023), and unlocking the potential to transfer these solutions from nature into improving crops.

What the next decade will hold for Rubisco research is hard to predict. This enzyme has provided the platform for crucial discoveries in protein folding and assembly which unlocked the potential to apply synthetic biology techniques such as directed evolution and to develop novel forms that are not present in nature. This applies directly to Rubisco but also informs discoveries and advances in understanding for other enzymes with important roles in nature. Application of machine learning to predict Rubisco kinetics is an emerging area (Iqbal *et al*., 2023). Interestingly, new developments that enable batch purification of the most abundant protein in plants, ­Rubisco, are now showing potential as an alternative food source (Pearce and Brunke, 2023).

Without doubt, research into the world’s most abundant enzyme has paved the way for many discoveries including protein folding assembly pathways, specificity of chaperonin complexes, stabilization of oligomeric proteins while crucial subunits are added, and the intricacies of regulatory mechanisms underlying the dynamics of plant metabolic responses to changes in the surrounding environment. Rubisco has been, and continues to be, an outstanding platform for pure basic research into understanding many biochemical properties of enzymes, driving creativity in biological sciences and innovations that push forward the frontiers of knowledge.
